# Duration and predictors of colonization with MDR *Escherichia coli* upon rehospitalization: a retrospective cohort study

**DOI:** 10.1016/j.infpip.2026.100584

**Published:** 2026-08-25

**Authors:** Jakob Thielmann, Robin Otchwemah, Frauke Mattner

**Affiliations:** a Chair of Hygiene and Environmental Medicine, Faculty of Health / Department of Human Medicine, University Witten/Herdecke, Cologne, Germany; b Institute for Hygiene, Cologne Merheim Medical Centre, University Hospital Witten/Herdecke, Cologne, Germany; c Central Department of Hygiene and Environmental Medicine, University Hospital Essen, University of Duisburg-Essen, Essen, Germany

**Keywords:** MDR *E. coli*, Colonization, Rehospitalization, Gastrointestinal diseases, Infection control

## Abstract

**Background:**

Multi-drug-resistant (MDR) *Escherichia coli* display a growing infection-control concern in hospitals, yet the duration of faecal carriage and risk factors for persistence are incompletely understood.

**Methods:**

In this retrospective cohort study, 217 patients with a positive rectal screening for MDR *E. coli* and a re-admission with repeated screenings were included. Time to clearance was analysed using Kaplan–Meier methods. Risk factors were determined with regression analysis.

**Results:**

At rehospitalization, 51.6% of patients remained colonized. Analysis estimated a median colonization duration of 9.26 months; nearly 20% remained colonized for several years. ICD-10 (International Statistical Classification of Diseases and Related Health Problems, 10^th^ Revision) category K (diseases of the digestive system) was associated with higher rate of persistence (odds ratio 2.07, *P* = 0.02).

**Conclusions:**

Colonization with MDR *E. coli* for a median of around 9 months underscores the relevance of control at re-admission. The association of gastrointestinal (GI) diseases with prolonged carriage suggests that targeted risk-based screening strategies may be appropriate. Approximately half of patients colonized with MDR *E. coli* remain colonized at first re-admission, and the median carriage duration is around 9 months. A minority may carry the organism for several years. GI diseases lead to a higher risk for prolonged colonization.

## Highlights

• MDR *E. coli* persist in 51.6% of patients at first re-admission.
• Median MDR *E. coli* colonization duration is ∼9 months.
• Gastrointestinal diseases (ICD-10 K) predict prolonged carriage.
• Comorbidities, age, and sex show no association with persistence.
• Findings advocate extended screening for prior MDR carriers.

## Background

Multi-drug-resistant *Escherichia* *coli* (MDR *E. coli*) have become an important cause of healthcare-associated colonization and infection [1]. Although the prevalence of MDR *E. coli* in the general German population is 5–6% [1], these organisms pose a significant threat in hospitals. Infected or colonized patients experience prolonged hospital stays and show increased mortality rates compared with patients with susceptible strains [2,3]. Therefore, infection-control policies in many hospitals mandate screening of patients with risk factors or known history of MDR, classified as 3MRGN (MDR Gram-negative bacteria, resistant to acylureidopenicillins, third-/fourth-generation cephalosporins, and fluoroquinolones) colonization, followed by isolation or contact precautions if screened positive. Unlike methicillin-resistant *Staphylococcus aureus*, for which decolonization protocols exist, there is no reliably effective decolonization therapy for MDR *E. coli*, and spontaneous clearance is unpredictable. Thus, understanding how long colonization persists is critical for guiding the duration of isolation precautions and the identification of patients upon readmission.

Because most MDR *E. coli* isolates in our cohort were expected to overlap with extended-spectrum β-lactamase (ESBL)–producing Enterobacterales, we compared our findings with the literature on ESBL-producing *E. coli*. Nevertheless, MDR/3MRGN colonization remained the primary outcome of our analysis.

Prior studies on the duration of carriage of EBSL-producing Enterobacterales, which include most MDR *E. coli*, have reported median durations ranging from a few months in hospital-based cohorts to over a year in long-term care settings, with generally shorter, transient colonization in community and travel-related contexts [[4], [5], [6], [7], [8], [9], [10]].

We conducted a retrospective cohort study of patients previously colonized with MDR *E. coli* to determine (1) the median duration of colonization upon rehospitalization and (2) predictors of persistent carriage at re-admission, according to the German ICD-10 (International Statistical Classification of Diseases and Related Health Problems, 10^th^ Revision). We hypothesized that patients with gastrointestinal (GI) diseases (ICD-10 chapter ‘K,’ e.g. chronic gastrointestinal (GI) diseases or abdominal surgeries) might have prolonged colonization, given that the GI tract is the reservoir for faecal carriage. The ultimate goal of this study was to inform hospital infection-control policies regarding the duration of precautions for MDR *E. coli* carriers and to identify patients who may benefit from individualized hygiene measures and monitoring at hospital re-admission.

## Methods

### Study design and setting

We performed a retrospective cohort study at two hospitals within the Cologne Medical Centre (a secondary-care and a tertiary-care hospital) covering the period from December 2019 to February 2025. Active surveillance of MDR organisms was implemented for high-risk areas, defined as intensive care units (ICUs) (surgical, internal medicine, neurologic including the stroke unit, pulmonary, and burn units).

Inclusion criteria were given as follows: (1) Initial colonization with MDR *E. coli*, documented by a positive screening culture obtained from an anal, rectal, perianal, or perineum swab during hospitalization or outpatient visit at one of the study hospitals, between December 2019 and February 2025, and (2) rehospitalization after the initial admission with a positive screening result, with a screening swab result available at the time of re-admission. This study exclusively examined patients without clinical evidence of MDR *E. coli* infection.

### Exclusion criteria

Exclusion criteria were given as follows: We did not analyse patients who had any positive MDR *E. coli* culture within 6 months prior to the initial screening, to exclude patients who already might have been persistent carriers from earlier episodes. Patients who did not have a microbiological screening result at the time of rehospitalization, e.g. due to a missed follow-up or screening on re-admission, were excluded. Originally, we identified 986 patients as positive for MDR *E. coli* in the study period; after excluding those with incomplete follow-up or screening, the final cohort comprised N = 217 patients for analysis.

### Microbiological methods

This study was based on routine infection-control screening for MDR *E. coli*. Rectal, anal, perianal, or perineal swabs were inoculated on to selective chromogenic media for MDR Gram-negative organisms and on to standard blood and MacConkey agar. Suspected colonies were identified by Matrix-Assisted Laser Desorption/Ionization Time-of-Flight mass spectrometry. Antimicrobial susceptibility testing was performed using automated systems (BD Phoenix™ and/or bioMérieux VITEK® 2 XL) and interpreted, according to contemporary European Committee on Antimicrobial Susceptibility Testing (EUCAST) clinical breakpoints for *E. coli*. The antibiotic panel included aminopenicillins and acylureidopenicillins (ampicillin, ampicillin/sulbactam, piperacillin, piperacillin/tazobactam), second- and third-generation cephalosporins (cefuroxime, ceftriaxone, cefotaxime, ceftazidime), carbapenems (ertapenem, imipenem, meropenem), an aminoglycoside (gentamicin), a fluoroquinolone (ciprofloxacin), cotrimoxazole, and tigecycline. MDR *E. coli* was defined as 3MRGN according to the German KRINKO (Kommission für Krankenhaushygiene und Infektionsprävention) classification, i.e. resistance to at least three of the four key antibiotic classes used for MRGN categorization (acylureidopenicillins, third-/fourth-generation cephalosporins, fluoroquinolones, and carbapenems).

### Data collection

For each patient, we recorded demographic data (age, sex), primary and secondary discharge diagnoses at initial admission and subsequently categorized the diagnoses according to the 22 ICD-10 groups (A to U). Additionally, we recorded the interval between the end of the index admission until re-admission, the length of hospital stay, and whether patients received care in high-risk ICUs (surgical, internal medicine, neurologic including the stroke unit, pulmonary, or burn units for severely burned patients) or on general wards.

The primary outcome was the persistence of MDR *E. coli* colonization at rehospitalization, which was achieved by a positive screening result at first re-admission after being tested positive during the initial stay. Conversely, clearance, describing the loss of MDR *E. coli*, was equivalent to one negative screening result at first re-admission. Due to the short length of stay at re-admission, mostly only a single screening swab result was available at this time point. For patients with multiple re-admissions during the data period, we considered the first re-admission with a screening as the point of outcome measurement. It should be noted that, in routine infection-control practice at our institutions, discontinuation of contact precautions generally requires three consecutive negative screening results.

### Statistical analysis

We used descriptive statistics to summarize the cohort. Categorical variables were compared by χ^2^ test as appropriate and continuous variables by Mann–Whitney *U* test.

For risk factor analysis, we performed univariate logistic regression to identify variables associated with persistent colonization (positive vs negative on re-admission). Variables tested included age (as a continuous variable), sex, and the presence of a K diagnosis (yes/no), and the presence of other major ICD-10 chapter diagnoses, as well as the total number of recorded diagnoses for the first admission. Results are reported as odds ratios (ORs) with 95% confidence intervals (CIs) and *P*-values. A *P*-value of < 0.05 was considered statistically significant. Variables with clinical relevance (K diagnosis, age, sex, total number of diagnoses, high-risk ward treatment, length of stay, and time between admissions) were subsequently entered into a multi-variable logistic regression model to assess independent associations with persistence. To estimate the duration of colonization, we employed Kaplan–Meier survival analysis treating ‘loss of colonization’ as the event of interest (date of discharge of first admission and date of re-admission). Patients who remained positive at the last follow-up or who had not yet been cleared by the end of the study were censored at the time of last positive culture. The Kaplan–Meier curve was used to derive the median time to clearance (with 95% CI). Statistical analyses were performed using Python.

## Results

A total of 217 patients met our inclusion criteria. The majority of patients were male (57.6%), while the median age of all patients was 63 years (Table I). Patients were treated either on general wards and/or in ICU (27.6%) or only on general wards (72.4%), with comparable distributions across both positive and negative groups at re-admission.

**Table I:** Study cohort including all patients who had been tested positive for MDR *E. coli* at least once and had been re-admitted at least once

Number of patients	217
Female	92 (42.4%)
Age (median, min/max)	63 years
	(14, 91)
ICU	60 (27.6%)
Non-ICU	157 (72.4%)
Length of stay (mean, SD)	16.04 d (23.96)
No. of diagnoses per patient (mean, SD)	15.15 (9.62)

MDR *E. coli*, multi-drug-resistant *Escherichia coli*; SD, standard deviation; ICU, intensive care unit.

Baseline characteristics of the patient cohort.

Detailed characteristics of the cohort are presented in Table I. At re-admission, 105 of 217 patients (48.4%) tested negative for MDR *E. coli* (classified as loss of colonization), while 112 patients (51.6%) remained positive. Numbers of primary and secondary ICD-10 diagnoses per patient are presented in Table II.

**Table II:** Diagnoses: number of main and secondary diagnoses per patient. ICD-10 chapters show patients with ≥1 diagnosis in the respective chapter; multiple chapters per patient possible

ICD-10 chapters	Patients with ≥1 ICD-10 code in chapter, n	ICD-10 chapters	Patients with ≥1 ICD-10 code in chapter, n
Z (Factors influencing health status and contact with health services)	187	T (Injury, poisoning and certain other consequences of external causes)	47
U (Codes for special purposes)	164	F (Mental, Behavioral and Neurodevelopmental disorders)	39
I (Diseases of the circulatory system)	157	M (Diseases of the musculoskeletal system and connective tissue)	29
E (Endocrine, nutritional and metabolic diseases)	154	S (Injury, poisoning and certain other consequences of external causes)	24
B (Certain infectious and parasitic diseases)	115	A (Certain infectious and parasitic diseases)	21
N (Diseases of the genitourinary system)	109	H (Diseases of the eye, adnexa, ear and mastoid process)	15
J (Diseases of the respiratory system)	101	C (Neoplasms)	15
D (Diseases of the blood and blood-forming organs and certain disorders involving the immune mechanism)	72	L (Diseases of the skin and subcutaneous tissue)	8
K (Diseases of the digestive system)	66	Q (Congenital malformations, deformations, chromosomal abnormalities, and genetic disorders)	5
R (Symptoms, signs and abnormal clinical and laboratory findings, not elsewhere classified)	65	O (Pregnancy, childbirth and the puerperium)	3
G (Diseases of the nervous system)	55	Y (External causes of morbidity)	1

Distribution of ICD-10 chapters among patients.

ICD-10, International Statistical Classification of Diseases and Related Health Problems, 10th Revision.

The interval between the initial positive admission and re-admission varied considerably. Although the mean time to re-admission was 6.19 months (± 8.33 months) and the median of 2.56 months, indicating data that were highly left-skewed. At the same time, few patients were re-admitted much later, with a maximum of 43 months. Univariate analyses are shown in Table III. There were no significant differences in sex distribution (*P* = 0.27) or age between patients who cleared colonization (negative group) and those who remained colonized (positive group, *P* = 0.94). Similarly, the distribution between ICU and general wards did not differ significantly (*P* = 0.23).

**Table III:** Univariate analyses comparing patients who remained colonized with MDR *E. coli* at re-admission and those who cleared colonization

Characteristic	Pos. at re-admission	Neg. at re-admission	*P*-Value	OR (95% CI)
Sex (%)				
Male	61 (54)	65 (61.9)	0.27	0.71 (0.41–1.22)
Female	52 (46)	40 (38.1)		1.41 (0.82–2.42)
Median age (min; max)	62.5 years (14; 91)	64 years (16; 88)	-	-
ICU	27 (45%)	33 (55%)	0.23	0.69 (0.38–1.26)
Non-ICU	85 (54.1%)	72 (45,9%)		1.44 (0.79–2.62)
Length of stay (mean, SD)	13.9 d (15.07)	18.32 d (30.66)	0.95	-
Diagnoses (mean, SD)	15.55 (9.85)	14.71 (9.38)	0.35	-
**ICD-10 chapters**	**Patients with ≥1 ICD-10 code in chapter, *N***			
Z	101	86	0.43	1.51 (0.54–4.22)
U	86	78	0.66	0.85 (0.42–1.72)
I	85	72	0.62	1.18 (0.61–2.28)
E	81	73	0.76	0.90 (0.47–1.72)
B	64	51	0.42	1.25 (0.72–2.19)
N	60	49	0.57	1.17 (0.67–2.04)
J	53	48	0.84	0.94 (0.54–1.64)
D	43	29	0.17	1.51 (0.84–2.70)
K	43	23	0.02	2.07 (1.13–3.80)
R	31	34	0.28	0.72 (0.40–1.30)
G	31	24	0.58	1.19 (0.64–2.22)
T	25	22	0.99	1.00 (0.52–1.92)
F	21	18	0.93	1.03 (0.51–2.08)
M	15	14	0.86	0.93 (0.42–2.05)
S	9	15	0.10	0.48 (0.20–1.17)
A	10	11	0.59	0.78 (0.32–1.93)
H	11	4	0.10	2.58 (0.79–8.39)
C	8	7	0.99	1.01 (0.35–2.89)
L	4	4	1.00	0.88 (0.21–3.60)
Q	3	2	1.00	1.33 (0.22–8.12)
O	1	2	0.60	0.43 (0.04–4.87)
Y	0	1	0.47	0.29 (0.01–7.21)

MDR *E. coli*, multi-drug-resistant *Escherichia coli*; Pos. at re-admission, positive at re-admission; Neg. at re-admission, negative at re-admission; OR, odds ratio; CI, confidence interval; ICU, intensive care unit; SD, standard deviation; ICD-10, International Statistical Classification of Diseases and Related Health Problems, 10^th^ Revision.

Clinical characteristics and ICD-10 chapter distribution by Re-admission status.

The cohort was divided into four quantiles based on the time to rehospitalization. No quantile showed significant association with MDR *E. coli* positivity at rehospitalization (quantile 1 showed median time of 0.30 months, *N* = 55, 50.9% positivity rate (pos.); quantile 2 showed median time of 1.61 months, *N* = 55, 56.4% pos.; quantile 3 showed median time of 4.2 months, *N* = 53, 54.7% pos.; and quantile 4 showed median time of 17.5 months, *N* = 54, 44.4% pos.). This analysis confirms that timing of re-admission does not predict colonization persistence across the distribution.

We compared patient characteristics between those who cleared colonization by the first follow-up (negative group) and those who remained colonized (positive group) (Table III). Interestingly, there was no significant difference in the overall comorbidity burden approximated by the number of diagnoses: both groups showed a median of 13 ICD-10 diagnoses. The average number of ICD-10 diagnoses was 15.55 in the positive group and 14.71 in the negative group, although the difference was not statistically significant (*P* = 0.14). These diagnosis counts include all primary and secondary ICD-10 codes recorded during the index admission, so that multiple comorbidities and procedure-related codes contribute to the total number per patient. Next, we examined the association of certain clinical factors with the likelihood of remaining colonized at re-admission. Our analysis revealed a significant association with diagnoses related to diseases of the digestive system, which are described as codes K00–K93 in the ICD-10 Chapter XI (Figure 1).

**Figure 1:**
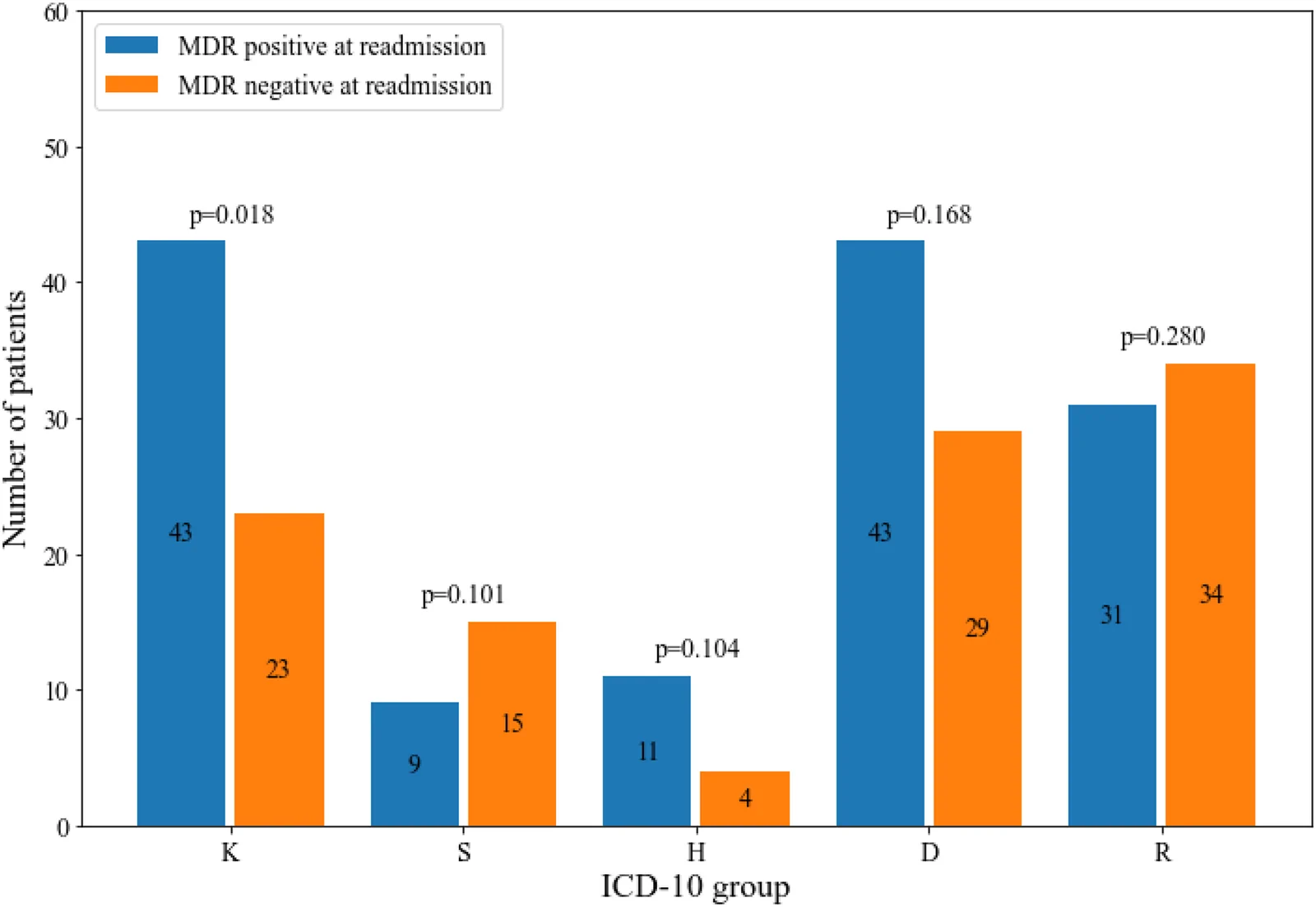
Association of ICD-10 factors and prolonged MDR *Escherichia coli* colonization. Only ICD-10 categories with a *P*-value <0.4 are shown. ICD-10, International Statistical Classification of Diseases and Related Health Problems, 10^th^ Revision; MDR *E. coli*, multi-drug-resistant *Escherichia coli*; K = Diseases of the digestive system, S = Injury, poisoning and certain other consequences of external causes, H = Diseases of the eye, adnexa, ear and mastoid process, D = Diseases of the blood and blood-forming organs and certain disorders involving the immune mechanism, R = Symptoms, signs and abnormal clinical and laboratory findings, not elsewhere classified.

Patients with any primary or secondary K diagnosis were more likely to remain colonized upon re-admission than patients without digestive system issues. For the persistence of MDR *E. coli* combined with a K diagnosis, we estimated an OR of 2.07 (95% CI 1.13–3.80), with a *P*-value of 0.02. Thus, 68% of patients with K diagnosis remained positive at follow-up (Figure 1). All other International Classification of Disease (ICD) codes assigned to different ICD-10 chapters seem to have no statistical relevance.

To explore independent predictors of persistent MDR *E. coli* colonization, we fitted a multi-variable logistic regression model including all clinically relevant covariates (presence of a K diagnosis, total number of diagnoses during the index admission, age, sex, treatment in a high-risk ward, length of stay, and the time interval between the first positive admission and re-admission). In this model, only the presence of an ICD-10 K diagnosis remained significantly associated with persistence (adjusted OR 2.12, 95% CI: 1.12–3.99; *P* = 0.02), indicating that patients with at least one digestive system diagnosis had roughly twice the odds of remaining colonized at rehospitalization compared with those without K diagnoses, independent of comorbidity burden, age, sex, ward type, length of stay, and re-admission interval. None of the other covariates showed a statistically significant association with persistent colonization; effect estimates for age, sex, total number of diagnoses, high-risk ward treatment, length of stay, and time between admissions were close to unity with wide CIs. Overall model fit was modest (McFadden’s pseudo-R^2^ 0.046).

We used Kaplan–Meier analysis to estimate the duration of colonization of MDR *E. coli*, accounting for the varying re-admission times. The graph shows the proportion of patients, who are still colonized since the initial positive MDR *E. coli* culture over time (Figure 2). The median is at 9.26 months or approximately 313 days and represents a 50% clearance of patients after initially being tested positive for MDR *E. coli*.

**Figure 2:**
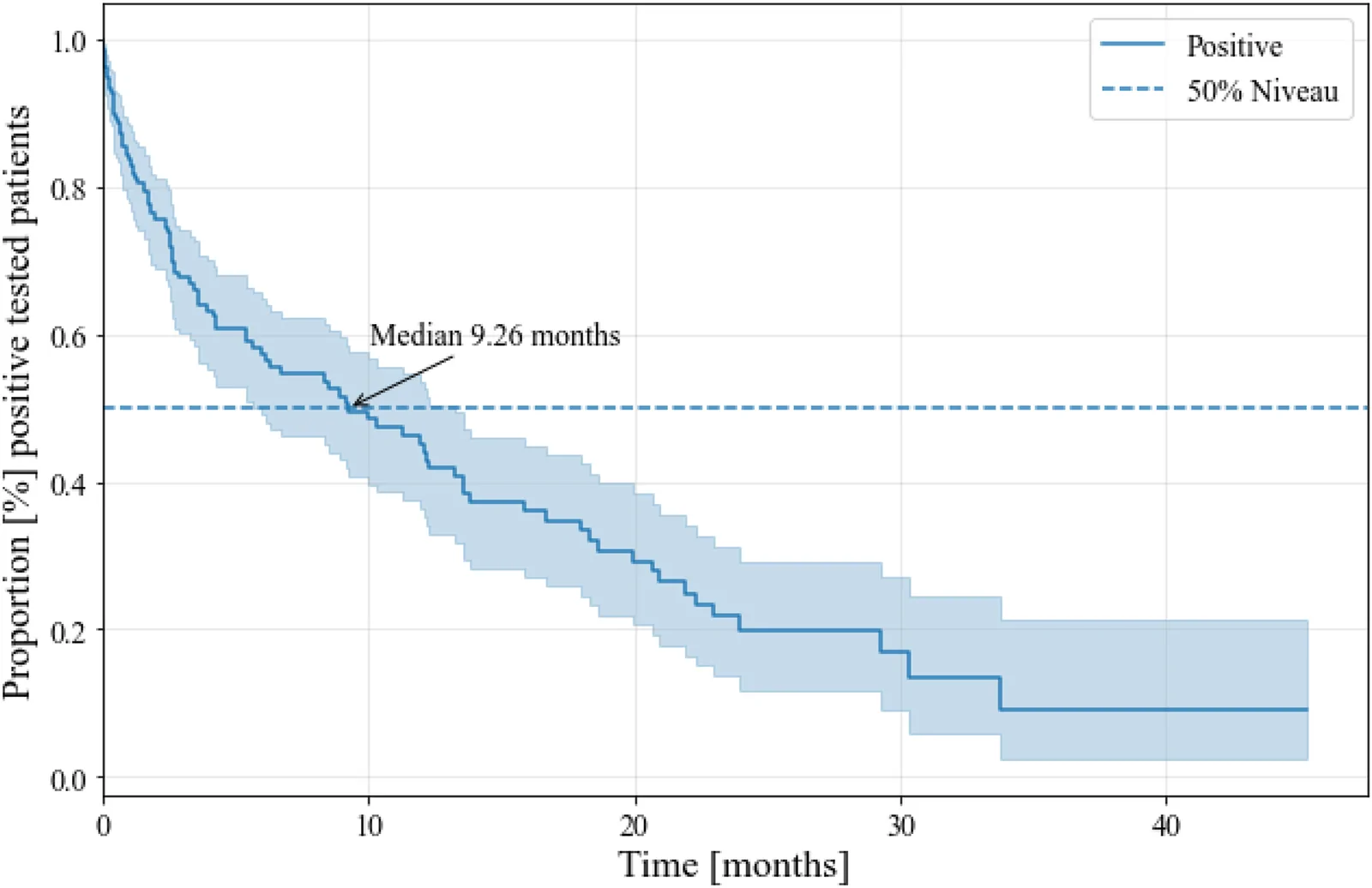
Estimating the time of clearance after colonization with MDR *Escherichia coli*. MDR, multi-drug-resistant *Escherichia coli*.

We compared the ICD codes assigned to patients who tested negative with those assigned to patients who tested positive for MDR *E. coli* upon re-admission.

The difference between patients with or without detection of MDR *E. coli* at re-admission was significant regarding the frequency of K diagnoses (Figure 2).

## Discussion

In this study, we investigated the colonization with MDR *E. coli* in a whole hospital patient population for over 4 years. We used re-admission data to characterize duration of colonization and identified factors associated with prolonged carriage. Although our cohort consisted of MDR *E. coli* carriers, several of the cited studies focused on ESBL-producing *E. coli*. This comparison is informative because ESBL production frequently co-occurs with multi-drug resistance, but the resistance definitions are not identical; therefore, our findings should reflect a broader MDR phenotype rather than ESBL carriage alone.

Colonization tends to persist for a long time in a substantial fraction of patients: the median duration was approximately 9 months, and about one in five patients remained positive for MDR *E. coli* for more than a year. This result indicates a longer time of colonization than previously reported in hospital-based studies of ESBL Enterobacterales carriage, which described a period of time between 4 and 7 months [4,6]. Zahar *et al.* reported a median faecal ESBL colonization of 132 days (≈4.3 months) in a mainly paediatric cohort [6]. Birgand *et al.* analysed patients re-admitted ≥3 months postdischarge in a 14-year surveillance with a median clearance of 6.6 months. Notably, 40% of patients in that cohort were still colonized after 6 months, and a follow-up study confirmed median carriage durations of 11–13 months among nursing home residents, with even longer persistence (up to 58 months in 12% of patients) reported after a hospital outbreak [4]. Because clearance was defined by a single negative swab result at re-admission, the estimated median duration reflects loss of detectable colonization rather than confirmed eradication and may therefore underestimate the true duration of carriage.

However, our estimate must be interpreted with caution, as it is derived exclusively from patients who were rehospitalized and re-screened. These patients are likely to differ systematically from non-rehospitalized carriers with regard to comorbidity burden, healthcare exposure, and possible antibiotic exposure. Therefore, the observed median duration may not reflect colonization dynamics in the broader population of MDR *E. coli* carriers. Differences in study design and study populations may therefore partly explain the variation between our findings and previously published data. A possible hypothesis for the prolonged colonization time of 9.26 months is the local incidence of certain bacterial strains in our hospital. Prior research in long-term care facilities showed that ST131 can be carried by patients for around 13 months, far exceeding the persistence of other strains [7]. Although we did not systematically genotype the isolates in our cohort, recent genomic epidemiology data from a German university hospital showed that ST131 dominated among MDR *E. coli* isolates, which supports the assumption that a relevant proportion of isolates in our cohort may also have belonged to this lineage. Frequent healthcare contacts and repeated hospital exposures may additionally have facilitated persistence or re-exposure in this patient population [11].

The most interesting finding of our study is the association between diseases of the digestive system (ICD-10 chapter XI) and the prolonged persistence of MDR *E. coli* in patients. K diagnoses were associated with significantly higher odds of persistent colonization. There are several hypotheses for this observation. First, underlying diseases of the digestive system might alter the gut microbiome or mucosal immunity in ways that favour MDR *E. coli*. For instance, patients with inflammatory bowel disease or those who underwent bowel surgery often have a disrupted microbiome. Due to the frequent exposure to antibiotics, the microbial competition is reduced, which again creates a niche for resistant micro-organisms such as MDR *E. coli* [12]. Another consideration is the suppression of gastric acid: many digestive system diseases are accompanied by treatments with proton pump inhibitors, which have been linked to higher risk of Enterobacterales colonization due to reduced stomach acidity barrier [13]. Our findings complement those of Lübbert *et al.* in travellers, in whom occurrence of travellers’ diarrhoea was a risk factor for acquiring ESBL *E. coli*. In contrast, travel-acquired ESBL-producing *E. coli* are typically cleared within weeks, with only ∼11% of travellers remaining colonized 12 months after returning from high-risk regions, underscoring how much shorter community-acquired colonization tends to be compared with our hospital cohort [9,10]. Future studies should ideally investigate differences in colonization duration between subgroups; however, our current dataset is unfortunately too limited to allow for such an analysis. Because multiple ICD-10 chapters were evaluated in parallel, the association with chapter K should be interpreted cautiously and regarded as hypothesis-generating, until confirmed in independent cohorts.

The adjusted association between ICD-10 K diagnoses and persistent MDR *E. coli* colonization suggests that the effect cannot be explained by age, sex, comorbidity burden, ward type, length of stay, or re-admission interval alone.

It is important to discuss that, while many patients eventually cleared MDR *E. coli* colonization, a non-trivial fraction did not clear within the observed period. Approximately 20% of patients remained positive. This raises the question of whether certain patients become indefinite carriers. Chronic colonization may result from an inability to mount an effective host response against the colonizing MDR *E. coli*. Another mechanism could be recolonization rather than relapse – a patient might clear the original strain but then reacquire another MDR *E. coli* from the environment, household, or healthcare setting [14]. In the context of our infection-control practices, however, this distinction may be irrelevant. Regardless of whether colonization is due to the same strain or a new one, the patient remains a carrier harbouring a resistant *E. coli* and posing a transmission risk if not isolated.

Despite these limitations, our study has important clinical implications. The prolonged median carriage of ∼9 months suggests that hospitals should maintain a high index of suspicion for known MDR *E. coli* carriers for at least a year after the initial detection. In practical terms, infection-control programmes may consider continuing to flag such patients in their alert systems for at least 12 months and require screening cultures at admission during that period. For example, in our hospitals, contact precautions for MDR *E. coli* carriers are only removed after three consecutive negative swabs. The fact that many patients were re-admitted quickly and more than 50% were still colonized at re-admission underscores that routine re-screening at rehospitalization is warranted. Failing to screen this population could lead to unrecognized carriers in the hospital, risking transmission.

Furthermore, identifying patients with risk factors such as digestive system diseases can help prioritize those requiring more stringent infection prevention measures. For instance, a patient who is known to carry MDR *E. coli* might benefit from tailored interventions. At a minimum, these patients should be handled with strict hygiene measures upon re-admission. Our findings also call for heightened stewardship of antibiotics in patients colonized with resistant bacteria, particularly those with K diagnoses – avoiding unnecessary antibiotics that might prolong carriage or selecting agents that spare gut flora when possible. In addition, preoperative antibiotic prophylaxis might be adjusted for patients with a K diagnosis.

### Limitations

First, our study is subject to selection bias because only patients with at least one rehospitalization and one repeated screening were eligible for inclusion (217/986 initially MDR *E. coli*–positive patients). This restriction means that our cohort represents a highly selected subgroup of colonized patients and may differ systematically from patients who were not rehospitalized, particularly with regard to comorbidity burden, cumulative healthcare exposure, and antibiotic treatment. Therefore, the estimated median duration of colonization should be interpreted as applying to rehospitalized MDR *E. coli* carriers in a tertiary-care setting and may not be generalizable to the broader population of colonized patients.

Second, clearance was operationally defined as a single negative screening swab result at first re-admission, which may have underestimated the true duration of carriage. Intermittent shedding and false-negative screening results are well recognized for MDR Enterobacterales. Therefore, some patients classified as cleared may in fact have remained colonized, and the reported median time to clearance should be interpreted as the time to loss of detectable carriage rather than definitive eradication.

Third, the retrospective design and reliance on routinely collected data limited the availability of potentially relevant clinical information. In particular, we lacked granular data on antibiotic exposure during and between admissions, including outpatient prescriptions, and therefore could not assess how interim antimicrobial treatment influenced persistence or clearance. Similarly, we did not have data on patient behaviours (e.g. healthcare facility residency, use of probiotics, or other exposures) that might modulate colonization.

Fourth, routine molecular typing was not performed as part of surveillance. Consequently, we could not determine whether persistent detection reflected prolonged carriage of the same strain or acquisition of a new MDR *E. coli* strain. Therefore, relapse and recolonization could not be distinguished.

Our work contributes to the broader understanding of MDR *E. coli* epidemiology in hospital patients. It helps to contextualize findings from outbreak or long-term care settings (with prolonged carriage) and from short-term travel or community settings (with brief carriage). The median clearance time of approximately 9 months observed in our hospital cohort lies between the estimates reported for community and long-term care settings, likely reflecting a mixture of both epidemiological contexts. This suggests that hospitals should remain vigilant for at least a year postcolonization for most patients and that the decay of carriers in the population is slow.

## CRediT authorship contribution statement

**Jakob Thielmann:** Writing – original draft, Visualization, Software, Methodology, Investigation, Formal analysis, Data curation. **Robin Otchwemah:** Supervision. **Frauke Mattner:** Supervision.

## Ethical approval

No ethics vote was required, as only existing data collected as part of routine surveillance in accordance with the applicable legal requirements under the Ordinance on Hygiene and Infection Prevention in Medical Facilities of the state of North Rhine-Westphalia in conjunction with Section 23 of the the German Infection Protection Act were evaluated. This was a retrospective study without direct patient contact.

## Funding sources

This research did not receive any specific grant from funding agencies in the public, commercial, or not-for-profit sectors.

## Conflict of interest statement

All authors declare that they have no known competing financial interests or personal relationships that could have influenced the work reported in this study.
